# An anti vimentin antibody promotes tube formation

**DOI:** 10.1038/s41598-017-03799-2

**Published:** 2017-06-15

**Authors:** Mathias Lindh Jørgensen, Carina Kjeldahl Møller, Lasse Rasmussen, Louise Boisen, Henrik Pedersen, Peter Kristensen

**Affiliations:** 1 Department of Engineering, Gustav Wieds Vej 10, Aarhus University, 8000 Aarhus C, Denmark; 2 Department of Molecular biology, Gustav Wieds Vej 10, Aarhus University, 8000 Aarhus C, Denmark

## Abstract

In recent years, there has been an increasing appreciation of the importance of secreted and extracellular proteins that traditionally have been considered as intracellular components. Vimentin is a highly abundant intermediate filament protein, and its intracellular functions have been investigated in a large number of studies. Recently, however, vimentin has been shown to take part in significant processes outside the cell. Our understanding of the functions of extracellular vimentin is, however, limited. In this study we demonstrate that a vimentin specific antibody, obtained by phage antibody technology, promotes tube formation of endothelial cells in a 2D matrigel assay. By binding vimentin, the antibody increases the tube formation by 21% after 5 hours of incubation. Addition of the antibody directly to cultured endothelial cells does not influence endothelial cell migration or proliferation. The enhanced tube formation can be seen for up to 10 hours where after the effect decreases. It is shown that the antibody-binding site is located on the coil 2 domain of vimentin. To our knowledge this is the first study that demonstrates an enhanced tube formation by binding vimentin in a 2D matrigel assay under normoxic conditions.

## Introduction

The intermediate filament protein vimentin exert important intracellular functions, regulating processes like cell migration and sustaining cell integrity. The importance of vimentin-mediated processes was underestimated for years, mainly because vimentin deficient (*vim* ^−/−^) mice initially were described as displaying virtually normal phenotypes and no apparent physiological defects were observed^1^. Eventually, more detailed studies on *vim* ^−/−^ mice revealed that vimentin has key functions in events of cell signalling, migration and adhesion^2, 3^. The 57 kDa type III intermediate filament protein, consists of a head-, rod- and tail domain and can be post-translationally modified. A large number of phosphorylation sites have been characterised, especially on the head domain where the phosphorylation status regulate filament assembly^4, 5^. Deamination of arginine residues in the head domain leads to citrullination, which may be important in inflammatory diseases such as rheumatoid arthritis^6^. Vimentin may be further modified by addition of N-Acetylglucosamine or act as a specific target for glycation^7–9^. In addition, proteolytic cleavage by specific caspases and calpain further modify vimentin^10, 11^. Apart from the influence of post-translational modification on filament assembly, the functional effects of modification still remain largely unknown. Possibly due to the high intracellular abundance of vimentin, the extracellular localisation and possible functions has only been described in the last 15 years. Initially, vimentin was found on the surface of activated macrophages, but more recently it has been found on the surface of more cells including circulating tumour cells^12–14^. How the vimentin-mediated processes are regulated remains elusive, and it seems that vimentin is a highly dynamic molecule with tissue specific functions^15–17^. Vimentin has been shown to take part in viral and bacterial infections and may be a key partner in neuronal regeneration^18–21^. It has been suggested that vimentin may serve as a potential anti-angiogenic target, however, results are contradicting^22–24^. As angiogenesis is an essential part of sustaining oxygen supply in health as well as in disease, potential anti-angiogenic targets have to be carefully evaluated to avoid severe adverse effects by the anti-angiogenic treatment^13^. The ability to form new blood vessels is diminished with age. To increase our cellular and biochemical understanding of this age related decline in angiogenesis, we have previously established long-term in cultivation of endothelial cells^25^. We established that the ability of the endothelial cells to form tube like structures when grown on matrigel decline when allowed to age in culture^25^. To identify change of endothelial cell expression profile that correlates with the loss of ability to form tubes, we applied the phage antibody technology as a discovery tool. The Tomlinson I+J library was applied to select single chain fragment variable (scFv) antibodies against proteins of ageing HUVEC cells by the phage display technology^26, 27^. One of these antibodies, named LOB7, displays pro-angiogenic features in 2D matrigel assays. We have identified vimentin as the protein target for this antibody. The blockage of functional epitopes combined with detailed mechanistic studies most likely will pave the way for increased understanding of extracellular vimentin and possibly provide new leads to treat human disease.

## Results

### Selection of the LOB7 scFv against membrane associated proteins from HUVEC

Based on an *in vivo* biotinylation protocol developed by the group of Dario Neri^28^, a selection of antibodies was performed against biotinylated proteins from HUVEC cells. In total 384 clones were picked and monoclonal phage antibodies were produced followed by sceening for their binding to HUVEC and HMEC-1 cells by phage antibody ELISA (Supplementary Fig. S1). One of the antibodies selected for further investigation was LOB7. Initial characterisation by ICC showed that LOB7 bound more to old ASF-2 (passage 52) than to young ASF-2 (passage 10) (Supplementary Fig. S1).

### LOB7 binds vimentin

**Figure 1:**
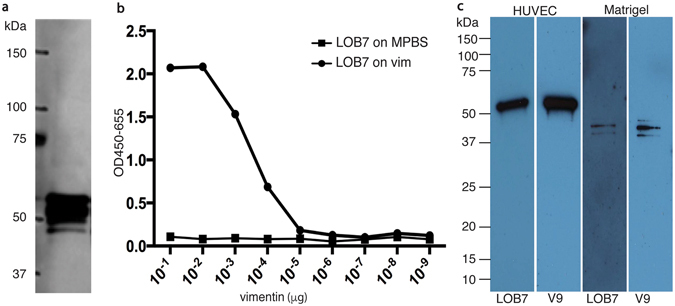
Characterisation of LOB7. Top panel: (**a**) The commercial mouse anti-vimentin V9 was used to identify vimentin in the sample of immuno-precipitated proteins. (**b**) LOB7 presented on phage was tested against vimentin in ELISA. A dilution series of vimentin was coated in the wells of an ELISA plate and detected using the same amount of LOB7 presented on phage. (**c**) Western blot using the commercial mouse anti-vimentin V9 and LOB7 respectively on cytoplasmatic extracts from HUVEC cells and extracts from the extracellular matrix (Matrigel).

The scFv holding a His tag motif was immobilised on Ni-NTA magnetic resin beads and used to precipitate proteins directly from sonicated HUVEC lysates. Precipitated proteins were separated by SDS-PAGE, visualised by silver stain, and bands of interest were excised from the gel and analysed by mass spectrometry (Supplementary Fig. S2). Vimentin was identified as the top hit from the mass spectrometry analysis (Supplementary Tabel S1). Some of the residual sample of precipitated proteins was analysed by western blot using the mouse monoclonal anti vimentin antibody V9 (Fig. 1a). This confirmed the presence of vimentin in the sample of proteins precipitated from HUVEC lysates by the use of LOB7. Accordingly, phage antibody ELISA was performed on serial diluted recombinant vimentin. A clear signal was observed (Fig. 1b). To further validate the binding of LOB7 to vimentin, a control ELISA was performed where laminin and skimmed milk powder was included as negative controls (Supplementary Fig. S3).

Additionally, the specificity of the LOB7 antibody was assessed by western blot analysis of HUVEC lysates and growth factor reduced matrigel. The anti-vimentin V9 antibody was used as a positive control. As can be seen, the proteins detected by LOB7 have the same apparent size as those detected by V9 (Fig. 1c).

**Figure 2:**
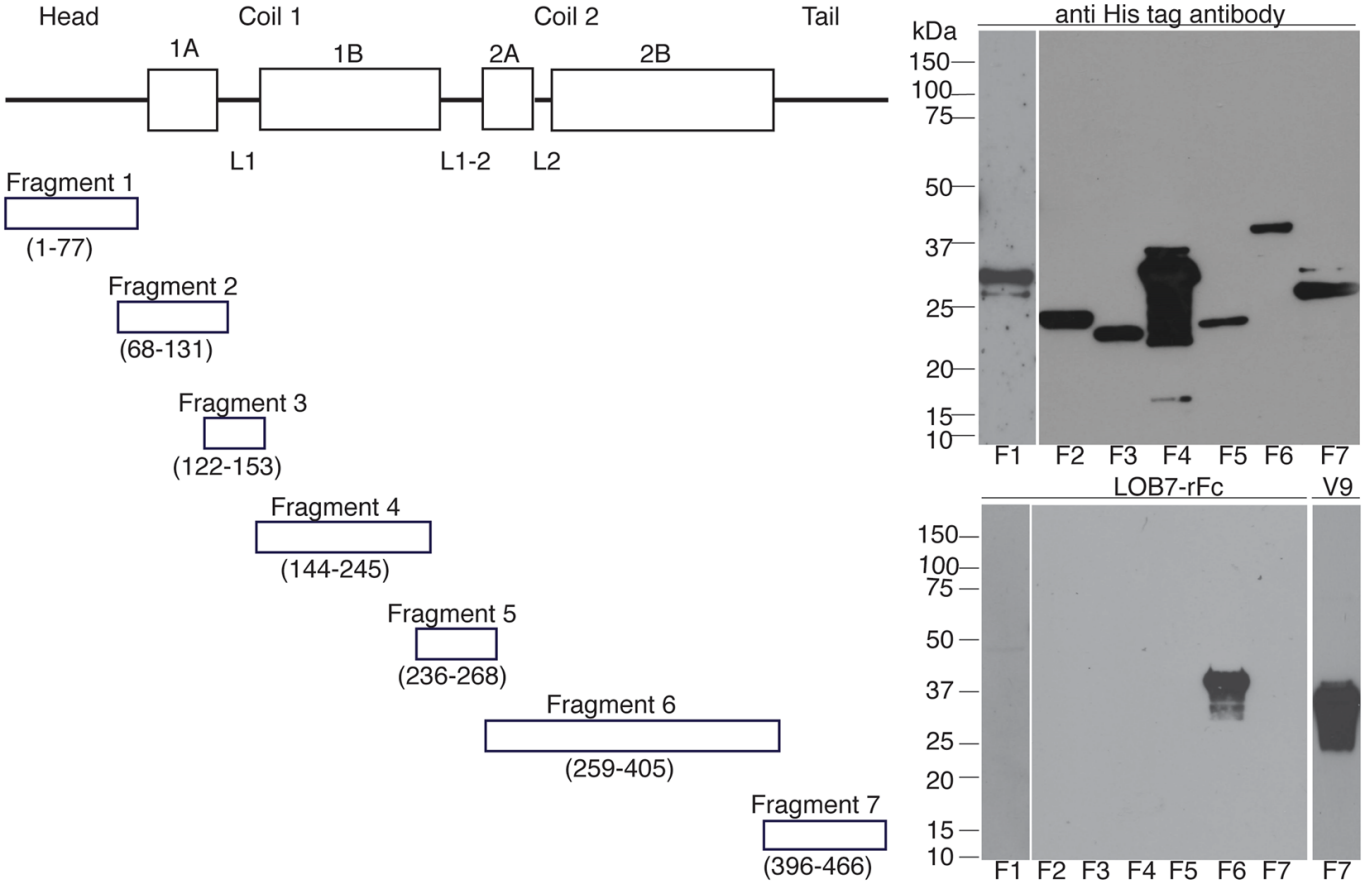
Testing specificity of LOB7 and V9 by western blotting. To test the specificity of LOB7 and the mouse monoclonal anti-vimentin antibody V9, seven fragments of vimentin were produced as fusions to the pagP proteins all carrying a His-tag. To the left, the fragments F1-F7 are schematically shown. In the right top panel, the seven fragments are detected by an anti-His antibody. In the lower right panel, LOB7 is seen to bind specifically to the F6 fragment. As there is no recognition of the F5 and F7 fragments, the epitope of LOB7 is between amino acid 268 and 396 of vimentin. The mouse monoclonal antibody V9 specifically bind to the F7 fragments, therefor the epitope for V9 is between amino acid 405 and 466.

To map the binding of the antibody, seven fragments covering different areas of vimentin were constructed by PCR and expressed in *E. coli* (Fig. 2). Long unstructured protein sequences, which are often seen in eukaryotic cells, are very susceptible to degradation^29^. Hence, to protect some of the intrinsically unstructured domains of vimentin against degradation, all seven fragments were expressed in conjugation with the pagP beta-barrel membrane protein^30^. It was shown that LOB7 binds to the coil 2 domain of vimentin, while the mouse anti-vimentin V9 binds to the tail region of vimentin.

### LOB7 promotes tube formation in a 2D matrigel assay

**Figure 3:**
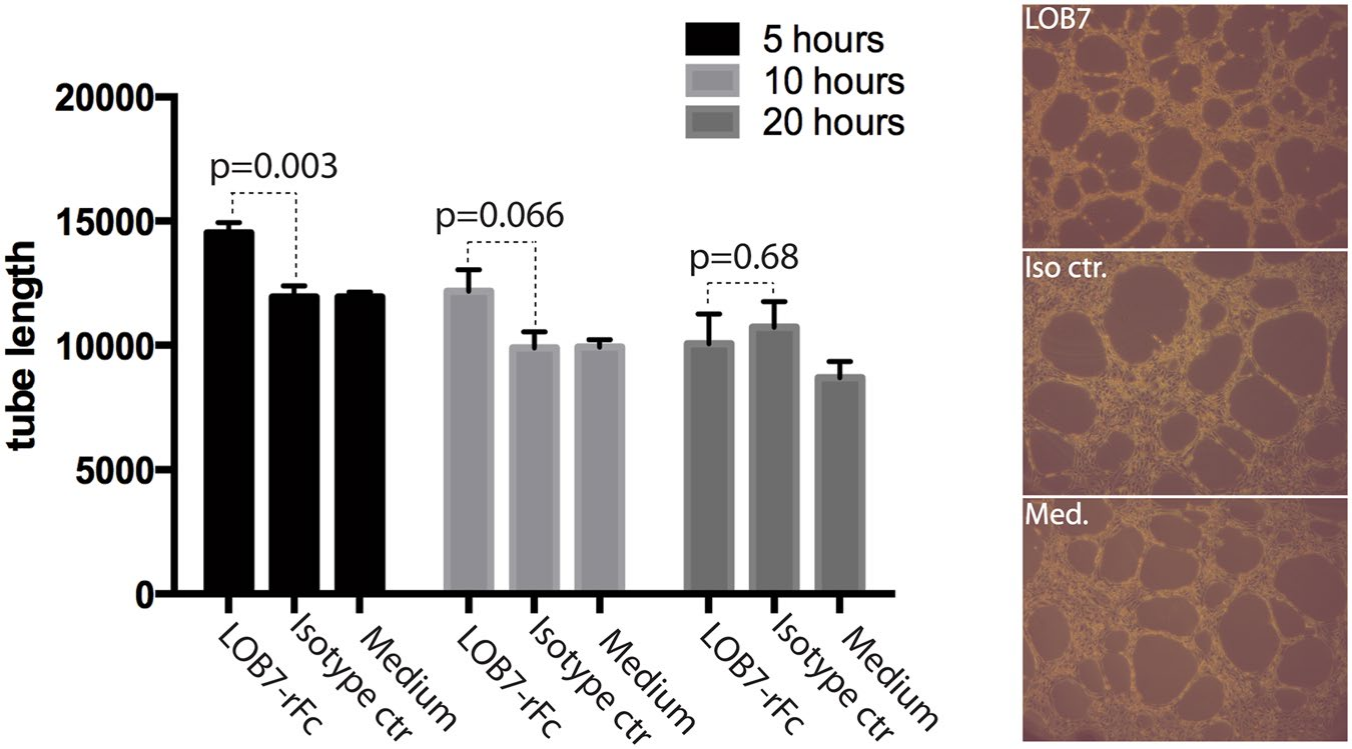
Tube formation using growth factor reduced matrigel in IBIDI angiogenesis slides. The tube length was measured as number of pixels using a MathWorks MatLab program. To the right, pictures are shown for 5 hours of incubation, from the top: LOB7, isotype control (anti HNE-α7), and medium only. Error bars: Standard error of the mean (SEM).

To assess the angiogenic effect of the anti vimentin antibody, five independent tube formation assays was performed with 5–7 replicates for each condition (Fig. 3). HUVEC cells were grown on growth factor reduced matrigel using three different conditions; 0.5 mg/mL of LOB7, 0.5 mg/mL of isotype control antibody, and no antibody.

After 5 hours, LOB7 induced a 21% increase of tube formation compared the control conditions (Fig. 3). Although not significant the effect could still be observed after 10 hours with an enhanced tubed formation of 23%, whereas the effect was absent after 20 hours. To examine whether LOB7 has an impact on migration, a scratch assay was performed. An effect on migration was not observed (Supplementary Fig. S4). Also, the influence on proliferation upon addition of the LOB7 antibody was examined, again without observing any significant enhancement of proliferation (Supplementary Fig. S4).

## Discussion

In this study, we describe a phage display scFv antibody selected against human endothelial cells grown in long-term culture. Mass spectrometry identified vimentin as the antigen of the LOB7 antibody. Increased expression of vimentin has previously been shown in ageing human cells in long-term culture^31^. Vimentin could be detected in the sample of precipitated proteins using a mouse anti-vimentin antibody (V9) in western blotting. These results were supported by ELISA tests of LOB7 against recombinant vimentin, and recombinant fragments of vimentin. The specificity of the antibody was confirmed by western blot analysis in which V9 and LOB7 were used for detection of vimentin in cell lysate and growth factor reduced matrigel. The antibodies revealed bands sharing the same apparent size. A discrepancy in apparent size of vimentin in matrigel compared to HUVEC lysate was observed (Fig. 1c). It is known that vimentin can differ in size depending on location^17^ and that caspase activity during apoptosis cleaves vimentin into sizes similar to those observed here^32^. The angiogenic effect of targeting vimentin remains unclear. Previously a vimentin specific phage display peptide was shown to promote the tube formation in 2-D matrigel assays by a 2-fold increase^24^. Even small amounts of this peptide induced the formation of tubes, however, the effect was only observed under hypoxic conditions. In contrast a study performed by Beijnum *et al*.^22^, not relying on 2-D matrigel assays, showed decreased tube formation by targeting vimentin in 3-D collagen assays, *in vivo* Chick chorioallantoic membrane assay, and in mice models by means of different anti vimentin antibodies^22^. In this study, we have presented an antibody that promotes tube formation under normoxic conditions in a 2-D matrigel assay. The mechanisms mediating the enhanced tube formation were not clarified. However, it was established that the LOB7 antibody does not have a direct influence on migration or proliferation of cultured endothelial cells. It can be hypothesised that the binding site of LOB7 stabilises the focal adhesion points between the cell surface and extracellular matrix. A process in which vimentin has key function^14^. We furthermore show that LOB7 recognises the coil 2 fragment of vimentin. It is of high functional relevance to gain more detailed insight to the epitope of LOB7. Furthermore, future experiments using *in vivo* models are imperative in order to understand and verify the effect of LOB7.

Vimentin overexpression relates to invasiveness and aggressiveness of cancer. Accordingly, vimentin can be regarded as a potential therapeutic target^13, 22, 33^. However, the dynamic roles of vimentin, as illustrated by the diversity of reported functions, indicate that adverse effects might arise by anti vimentin-based treatment. This is to our knowledge the first study demonstrating enhanced angiogenesis in a 2-D matrigel-based assay by targeting vimentin under normoxic conditions.

## Materials and Methods

### Cell Cultures

HUVEC cells were grown in EGM Bulletkit medium (cc-3124, Lonza) in tissue culture flasks coated with 0.2% gelatin. ASF-2 cells were grown in DMEM (Lonza) with 10% fetal bovine serum (Thermo Scientific), 100 U/mL penicillin and streptomycin (Lonza) at 37 °C, 5% CO2 and 95% humidity. Cells were detached from the tissue culture flasks by trypsination with Trypsin EDTA (Lonza). Cell were passaged 1:4 at approximately 80% confluence.

### Selection on biotinylated membrane proteins

Cells grown to subconfluency were labelled with EZ-link Sulfo-NHS-LC-Biotin (Pierce Biotechnology,) according to manufacturer’s instructions but adapted to adherent cells. Briefly, the cells were washed trice with cold PBS, labelled for 30 minutes at RT with 2 mM biotin. Labelling was quenched with 1 mM cold Tris-HCl, pH 7.4, washed once with Tris-HCl and twice with cold PBS. The cells were scraped in cold lysis buffer (20 mM Tris-HCl pH 7.4, 5 mM EDTA, 150 mM NaCl, 1% NP40, 10% glycerol) on ice and then lysed on ice for an additional 30 minutes.

Biotin-labelled lysate was coupled to 20 µl streptavidin coated magnetic beads (Roche Diagnostics) on a rotating wheel for 30 minutes at RT. Unbound lysate was removed using a magnetic concentrator and the beads washed twice with lysis buffer and once with M-PBS. Tomlinson I+J libraries were added in M-PBS and incubated on the rotating wheel for 1½–2 hrs at RT. Beads were washed 15 times with PBS-T and 15 times with PBS with 2 minutes incubation each time. After washing, the remaining phages were eluted by adding 100 μl trypsin in PBS (1 mg/ml). After 10 minutes the eluent was infected into TG-1 (OD_600_ 0.5) and spread on TYE plates with 100 μg/ml Ampicillin and 1% (W/V) Glucose. Plates were incubated at 30 °C overnight.

### Phage-ELISA on whole cells

For cell screenings, endothelial cells (HUVEC and HMEC-1) were seeded in 96-well cell culture plates, 10,000 cells/well in 100–200 µl of growth medium, and cultured for 1–4 days. After two times wash in PBS, the plates were either used directly or dried at 37 °C and frozen at −80 °C. As background controls, 100 µl/well of the appropriate growth medium was added to fresh 96-well cell culture plates and incubated overnight. After two times wash in PBS, the medium plates were used directly.

Coated plates were blocked in 2% M-PBS 1–2 hrs, washed 2–3 times in PBS and incubated with 50 µl phage solution and 50 µl MPBS 1–2 hrs. Plates were washed 2–3 times in PBS-T and incubated with horseradish peroxidase (HRP)-conjugated anti-M13 (GE Healthcare) diluted 1/5000 in MPBS for 1 hr. After 2–3 times wash in PBS-T, plates were developed using TMB (tetra-methylbenzidine, Invitrogen). Reaction was stopped with 1 M H_2_SO_4_ and absorbance was read at 450 nm with 655 nm as reference.

### SDS-PAGE and Western Blot

Proteins were separated on Criterion XT 12% Bis-Tris precast gels or for immunoprecipitated samples on Criterion XT 4–12% Bis-Tris precast gradient gels according to outlines of manufacturer (Bio-Rad). Samples for SDS-PAGE were boiled in XT sample buffer including XT reducing agent. In general, the separated proteins were blotted to 0.2 uM nitrocellulose membranes using the Trans-Blot^®^ Turbo™ Transfer System following the outlines of manufacturer (Bio-Rad). The membranes were blocked 1 hr in 2% marvel skimmed milk in phosphate buffered saline (MPBS). After and between incubations with primary and secondary antibodies, the membranes were washed in PBS for 3 × 5 min. The Amersham ECL Prime Western Blotting Detection Reagent (GE Healthcare) was used in combination with an HRP-conjugate for a chemiluminescent signal. The signal was detected on a MG-SR Plus x-ray film (Konica Minolta) and developed in the dark using an AGFA Curix 60.

### Silver stain

Gels were fixed for 1 hr or o/n in fixing solution (50% EtOH, 12% AcOH, 0.0185% formaldehyde). Fixed gels were washed three times in 35% EtOH followed by pre-treatment for 1 minute in 0.02% Na_2_S_2_O_3_. After two quick washes in demineralised H_2_O (dH_2_O), the gels were incubated in freshly prepared silver solution (0.2% AgNO_3_, 0.076% formaldehyde) on ice, 20 minutes. The gels were quickly washed in dH_2_O and incubated with developing solution (6% Na_2_C0_3_, 0.05% formaldehyde, 2% pre-treatment solution) until proteins were sufficiently visualised. Development was stopped by neutralisation with fixing solution.

### Expression of LOB7

LOB7 scFv expression was conducted directly from the phagemid in BL21. Expression was induced at OD_600_ 0.5 using 1 mM IPTG and incubated o/n at 30 °C and 200 RPM. The LOB7 scFv was purified from bacterial BL21 pellets of 2 L Terrific Broth culture as outlined in QiaExpressionist^TM^ fifth edition (p.82–83) using Ni-NTA Superflow Beads (Qiagen). Hydrophobic interaction chromatography (HIC) was included as an additional purification step. IMAC purified scFv were supplemented with 5 M NaCl to a final concentration of 2 M. The samples were loaded onto a HiScreen Butyl-FF HIC column (GE Healthcare). Proteins were eluted in a gradient of NaCl from 2 M – 0 M in 50 mM Tris-HCl, pH 8.0. Antibody containing fractions were pooled and concentrated using Vivaspin 6 columns, 10.000 kDa cutoff (Sartorius Stedim Biotech, Goettingen, Germany). As the scFv LOB7 did not express well in *E. coli*, the expression system was changed to *Leishmania tarentolae* T7-TR^34^. This was roughly performed as described in Jørgensen *et al*.^34^ but with a few exceptions. In short, two times 250 mL cultures were inoculated 1:10 in 1 L baffled flasks and incubated three days. Additional Hemin (1:1000) was added after 24 hrs of expression. The supernatants were harvested by centrifugation at 3000 × g for 15 min and sterile filtered (0.2 μm). Next, sterile filtered PBS was added to the supernatants to a final volume of 1 L and the pH was adjusted to 7.0. The purification was performed by protein A affinity chromatography using a 1 mL HiTrap Protein A HP column (GE Healthcare) in combination with ÄKTA Start (GE Healthcare). The outlines of the manufacturer were followed.

### Immunoprecipitation from HUVEC lysates

Preparation of cells was performed as outlined in QiaExpressionist^TM^ fifth edition (pp.86–87), and the immunoprecipitation was conducted as outlined in Qiagen Ni-NTA Magnetic Agarose Beads Handbook (pp.26–28). HUVEC cells from five TC75 flasks were harvested by mechanical detachment in PBS, pooled and centrifuged at 1000 × *g* for 10 min at 4 °C. The cell pellet was resuspended in 250 μL IP lysis buffer (50 mM NaH_2_PO_4_, 300 mM NaCl, 10 mM imidazole, 0.05% Tween 20, pH 8.0) and protease inhibitor cocktail (P8340, Sigma-Aldrich). Then, cells were lysed by sonication using 6 × 15 sec bursts at 75 w, including 10 sec cooling period between each burst. Debris was pelleted at 10.000 × g at 4 °C for 10 min. His tagged LOB7 scFv (50 ug) was incubated with 50 μL of Ni-NTA magnetic agarose beads as outlined in protocol. For each IP reaction, 50 μL of soluble lysate was diluted factor 10 in interaction buffer (50 mM NaH_2_PO_4_, 300 mM NaCl, 10 mM imidazole, pH 8.0) before incubation with Ni-NTA magnetic agarose beads to pre-clear the lysates for endogenous proteins carrying His motifs. Eluted samples were analysed by silver stain. Proteins bands that did not appear in the controls (beads and lysate only, beads and antibody only) were send for MS analysis. The MS was performed by Professor Rong Zeng (Shanghai Institute for Biological Sciences, Chinese Academy of Sciences, Shanghai, China). The peptides identified from the analyses were blasted against human and *E. coli* databases using the Sequest data analysis software to identify the proteins they were derived from. The search and data validation in the form of a list of hits can be found as Supplementary Tabel S1.

### Phage antibody ELISA against vimentin and laminin

Phages were produced and Phage antibody ELISA was roughly performed as outlined in the Tomlinson (I+J) protocol. Recombinant vimentin (NeoMarkers) was coated in a 96-well NUNC ELISA plate in decreasing amount ranging from 0.1 μg to 10^−9^ μg. Alternatively, 1 μg vimentin and 1 μg Laminin-1 (Sigma-Aldrich) were coated. The plate was blocked 1 hr at RT in 2% MPBS. The phages were incubated in 100 μL 2% MPBS holding 10^10^ phages/mL for 2 hrs. HRP-conjugated anti-M13 (GE Healthcare) was used 1:5000 and incubated 1.5 hrs at RT. For a chemiluminescent reaction, 100 μL TMB (Invitrogen) was added. The reaction was terminated using 50 μL 1 M H_2_SO_4._ The absorbance was measured at OD 450 nm with 655 nm as reference in a plate reader (BIORAD, model 550).

### LOB7 for detection of vimentin in HUVEC lysates and growth factor reduced matrigel

The Fc fused LOB7 was used in a 5 μg/mL concentration for detecting vimentin in HUVEC lysates and growth factor reduced matrigel. HUVEC cells harvested from a TC 25 culture flask was lysed using Cell LYTIC M (Sigma Aldrich) according to manufacturer. Growth factor reduced matrigel was thawed and diluted in precooled PBS by factor 10 and 15 μL of this sample was mixed with 5 μL pre-cooled XT sample buffer. The samples were boiled for 5 min. As a positive control, mouse monoclonal anti-vimentin V9 (Sigma Aldrich) was used (1 ug/mL). The primary antibodies were incubated with the blots o/n. For detection of LOB7 and V9, anti-rabbit HRP and anti mouse HRP (DAKO) were incubated with the blots for 1 hr at RT in a 1:1000 dilution.

### Mapping the binding of LOB7

Seven different DNA fragments of vimentin were inserted inserted into the pETpagP vector^30^. The seven different vimentin DNA fragments (Fig. 2) were produced by PCR amplification of human vimentin cDNA (Source BioScience). Unless otherwise stated all buffers, enzymes, and reagents used were purchased from ThermoFisher. Using DreamTaq mastermix and primers listed below in Table 1, fragments 1, 3, 6 and 7 were flanked with AlwNl restrictions sites. The PCR products were separated by agarose gel electrophoresis using 1% gel grade agarose (Sigma Aldrich) and SYBR Safe. Next, the correct sized bands were identified by UV light exposure and excised from the gel. The DNA was then extracted by the use of GeneJET Gel Extraction kit. The extracted DNA fragments and the pETpagP vector were then cleaved using AlwNI as outlined by the manufacturer. The cleaved fragments were separated by agarose gel electrophoresis as just described. Ligations were performed with T4 ligase as outlined by the manufacturer. The ligations were concentrated using DNA Clean & Concentrator-5 kit (ZYMO Research) and transformed into *E. coli* XL1 (Stratagene) by electroporation. The transformed XL1 bacteria were plated on agar plates containing 100 µg/mL ampicillin and 1% glucose. Colonies were grown in overnight cultures for DNA purification. DNA was purified using GeneJet plasmid Miniprep. Correct insertion and orientation of the fragments was confirmed by sequencing at Eurofins Genomics (Germany). The fragments 2, 4, and 5 could not be inserted by AlwNI restriction cloning as they all hold an AlwNI restriction site in their sequence. Accordingly, these fragments were PCR amplified with primers shown below and inserted into the pETpagP vector by *in vivo* assembly (IVA) cloning^35^.

**Tabel 1:** List of primers used for amplifying fragments of vimentin.

F1 frw	5′ TTTCAGATGCTGGGTGGTTCTGGTCTGGTTCCGCGTGGATCTATGTCCACCAGGTCC 3′
F1 rev	5′ GAGCAGCATCTGCACCCCGGGCACGCTGCTCC 3′
F2 frw	5′ GGTGGTGGTGGTGCAGGATCTTATTCTGCTGCTCCA3′
F2 rev	5′ TCCGCGTGGATCTGTGCGCCTGCGGAGC 3′
F3 frw	5′ TTTCAGATGCTGGGTGGTTCTGGTCTGGTTCCGCGTGGATCTCGCTTCCTGGAGCAGC 3′
F3 rev	5′ GAGCAGCATCTGCTCCTCCTCGTAGAGGTC 3′
F4 frw	5′ GGTGGTGGTGGTGCAGCTCCTGGATTTCCTCTTCG 3′
F4 rev	5′ TCCGCGTGGATCTTCGCGCCTGGGGGAC 3′
F5 frw	5′ GGTGGTGGTGGTGGGCAGCCGTGAGGTCAG 3′
F5 rev	5′ TCCGCGTGGATCTAAACTCCACGAAGAGGAAATCCAGG 3′
F6 frw	5′ TTTCAGATGCTGGGTGGTTCTGGTCTGGTTCCGCGTGGATCTGATGTTTCCAAGCCTGACC 3′
F6 rev	5′ GAGCAGCATCTGTTCCAGCAGCTTCCTGTAGG 3′
F7 frw	5′ TTTCAGATGCTGGGTGGTTCTGGTCTGGTTCCGCGTGGATCTGAGATTGCCACCTACAGG 3′
F7 rev	5′ GAGCAGCATCTGTTCAAGGTCATCGTGATGCT 3′

The pETpagP vector was changed for IVA coning using the primers 5′ AGATCCACGCGGAACCAGAC 3′ and 5′ CACCACCACCACCACCACT 3′. All constructs were transformed into *E. coli* BL21 (Stratagene) and expressed in small scale. In brief, 50 mL cultures were inoculated 1:100 in Terrific Broth media holding 100 µg/mL ampicillin and grown until OD_600_ 0.5 where the expression was induced by 1 mM IPTG (Sigma Aldrich). The expression was performed overnight at 30 °C and 200 RPM. The cells were then spun down at 5000 g for 30 minutes at 4 °C and the supernatants were discarded. Cell pellets were then resuspended in 20 mM Tris-HCl pH 8.0 holding 8 M urea, 500 mM NaCl, 5 mM imidazole, and 1 mM DTT. Next, lysis was conducted with a SONOPLUS ultrasonic homogenizer (Bandelin) with settings 50% pulses of 20 kHz and 28 W. The cells were sonicated in 3 × 3 minutes including 3 minutes on ice between each round. Finally, lysed cells were centrifuged at 4 °C and 15.000 g for 30 minutes. The fragments were detected in the supernatants by western blot, conducted as described above.

### Immunocytochemistry

Performed as described in Jørgensen *et al*.^34^.

### Angiogenesis Assay

Matrigel Matrix GFR (Corning) was added to μ-Slide Angiogenesis (Ibidi) as described in application notes 19 and 27 for IBIDI μ-Slide Angiogenesis. HUVEC cells (passage 3–5) were grown to 80% confluence, trypsinated with trypsin-EDTA (Gibco by life technologies, Thermo Scientific) and centrifuged at 500 × *g* for 5 min. Cells were resuspended in growth medium and 11.000 cells/well were seeded. Antibodies, thoroughly dialysed against PBS, were used in a 0.5 mg/mL concentration. The isotype control anti HNE-α7 was produced in the same system as LOB7^36^. Volumes were adjusted to 50 μL/well using PBS. The slides were incubated at 37 °C, 5% CO_2_, and 95% humidity. Two pictures of each well were taken every 5, 10 and 20 hrs with a AxioCamERc5s camera (Zeiss) through a Primo Vert microscope (Zeiss) using 4× magnification and phase contrast Ph0/0.4 phase. A customary software package written in MathWorks Matlab (R2015a) was used to measure the tube lengths (s1).

### Statistics

Assuming equal variance a two-tailed student’s t-test was applied for comparison of the different conditions used in the angiogenesis assay. Results were considered significant if P < 0.05. This statistical test is recommended for analysis of tube formation assays (Application note 27, Data Analysis of Tube Formation Assays, Ibidi).

### Proliferation and migration assay

The proliferation assay was performed with the 5-Bromo-2′-deoxy-uridine Labeling and detection kit III (Sigma Aldrich) relying on BrdU incorporation upon cell proliferation. Four independent proliferation experiments were performed, each with three replicates of each of the three conditions. Pre-coated with 0.1% gelatine, wells of a 96-well cell cultivation plate (SARSTEDT) were seeded with 10.000 HUVEC cells, passage 5–10. The cells were grown in EGM media (LONZA) for 6 hours before adding BrdU and rabbit Fc conjugated antibodies (0.1 mg/mL). After 16–20 hours, the proliferation was measured according to the outlines of the 5-Bromo-2′-deoxy-uridine Labeling and detection kit III.

Four independent scratch experiments were performed, each with three replicates of each of the three conditions. The scratch assay was performed in 24-well plates (SARSTEDT), each well was coated with 0.1% gelatine. To be able to take pictures of the same area at each time point, a line was drawn on the backside of each well prior to the experiments. HUVEC cells in passage 5–10 were seeded (70.000/well) and grown overnight in EGM. Scratches were made horizontally across the wells using a p200 pipette. The cells were then washed twice in DPBS before adding EGM holding relevant rabbit Fc conjugated antibodies (0.1 mg/mL). Pictures were acquired with an AxioCam ERc 5 s camera every 1.5 hours for 6 hours. All images were blinded and analyzed using the ImageJ analysis software. Each image was analysed by manually tracing the leading edges of the scratched area to obtain the area of the wound. Acquisition of pictures and calculation of migration rate was performed according to the outlines of IBIDI application note 30 – Data analysis of wound Healing Assays.

## Electronic supplementary material

Supplementary information
