# Supplementary material for: An anti vimentin antibody promotes tube formation
Source: Sci Rep. 2017 Jun 15;7:3576. doi: 10.1038/s41598-017-03799-2 (PMC5472577; doi:10.1038/s41598-017-03799-2)
Supplement: Supplementary file 1 — Supplementary information [file 41598_2017_3799_MOESM1_ESM.pdf]

SUPPLEMENTARY INFORMATION

An anti vimentin antibody promotes tube formation

Mathias Lindh Jørgensen[1], Carina Kjeldahl Møller[2], Lasse Rasmussen[2], Louise Boisen[2], Henrik Pedersen[1], Peter Kristensen[1*]

Supplementary Figure S1

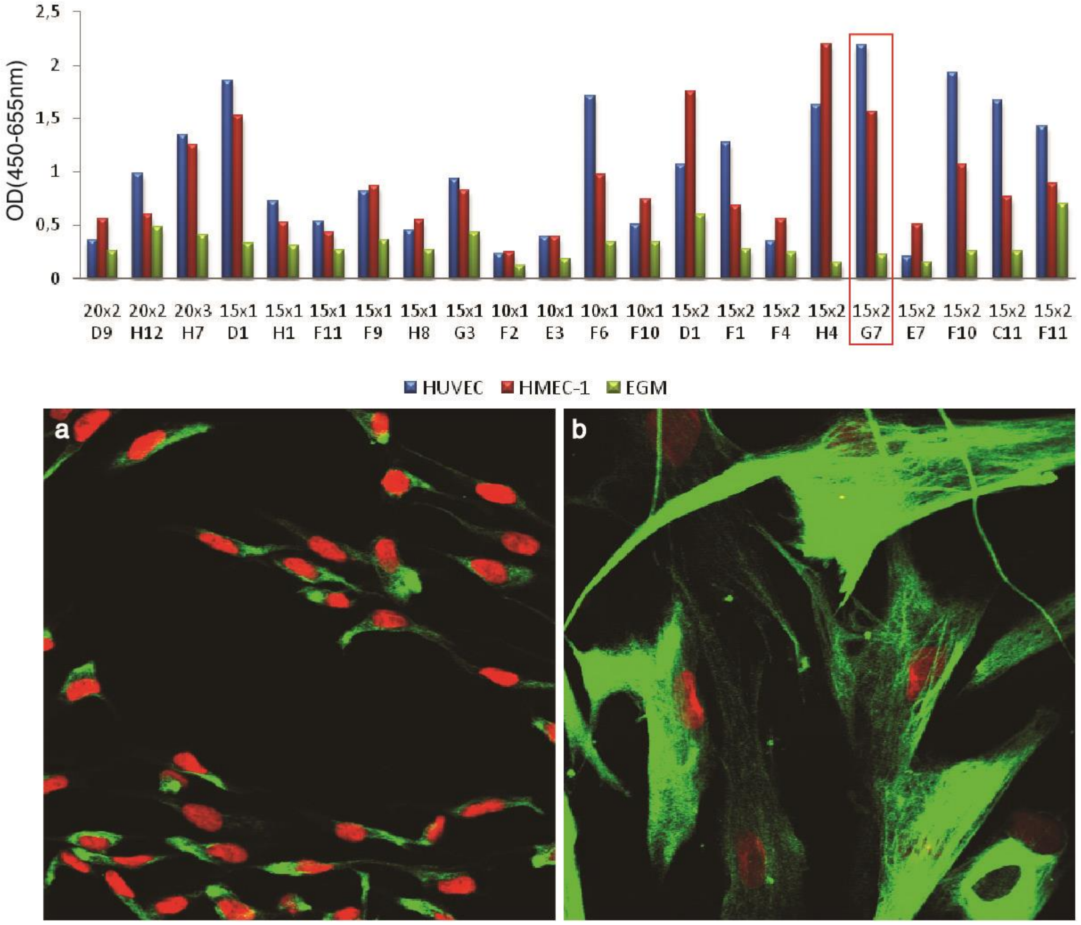

Supplementary Figure S1 Top: 384 colonies were picked for screening against endothelial cells. The screening of a subset of these clones is shown here. The LOB7 clone, formerly named 15x2 G7, is marked by a red rectangle. This initial screening showed that LOB7 binds to HUVEC and HMEC-1 cells. **Bottom**: Immunocytochemistry was performed on ASF-2 cells in passage 10(picture a) and passage 52(picture b). Nuclear stain is shown in red and antibody stain is shown in green. It was seen that the target of LOB7 was highly expressed in old cells (picture b). Retrospectively, this makes sense as old cells up regulate their expression of vimentin.

Supplementary Figure S2

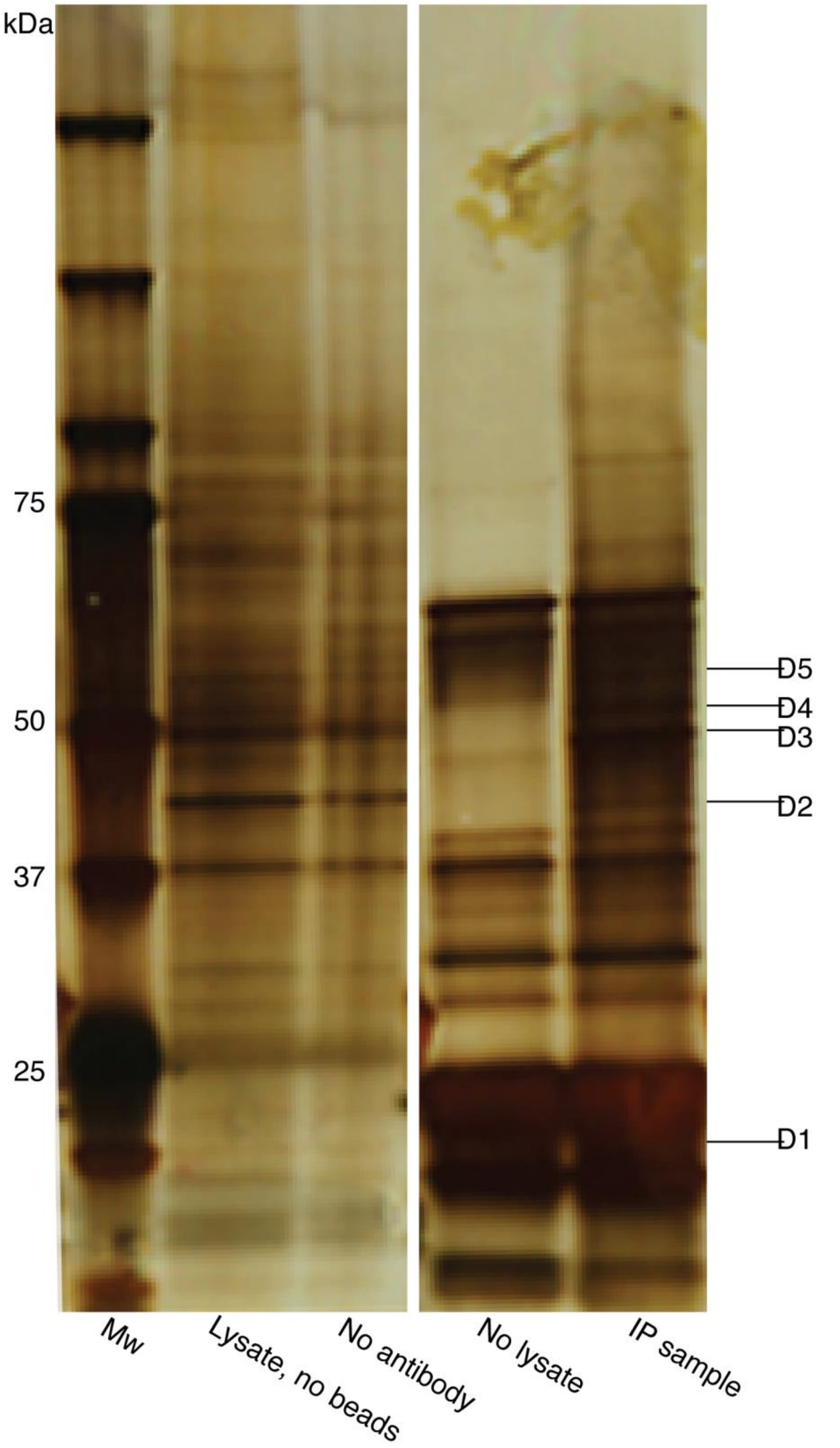

Supplementary Figure S2 IP was performed using the LOB7 scFv antibody. Different controls were included to identify possible precipitated proteins. From the left: HUVEC cell lysate without beads, lysates with beads but without antibody, beads with antibody but without lysates, and the IP sample with beads coated with antibody and incubated with cell lysate. Five bands, D1-D5, were excised and send for MS analysis

Supplementary Table S1

Ranked proteins from MS analysis performed by Professor Rong Zeng (Shanghai Institute for Biological Sciences, Chinese Academy of Sciences, Shanghai, China). Five bands (D1-D5) were distinguished from the control samples (Supplementary Fig S2), excised from the gel and send for MS analysis. Vimentin was the top hit. An *E. coli* protein was further identified as a top hit, which is a contamination from the antibody purification from the *E coli* expression culture. Keratin 31 and Elongation factor 1-α were also identified, however, these are commonly seen contaminants in IP/MS experiments.

Rank	Identified protein	Number of peptides identified	Number of unique peptides identified	MW	PI	Origin of peptides (excised band from gel)
1	VIM Vimentin	18	6	53651.88	5.06	D4, D5
2	*E. coli* protein	57	4	20852.79	4.86	D1, D2, D3. D4
3	EEF1A1 Elongation factor 1-alpha 1	12	4	50140.69	9.1	D3
4	KRT31 51 kDa protein	11	4	50668.35	4.97	D4, D5
5	DCD Dermcidin precursor	2	1	11283.86	6.09	D3

Supplementary Figure S3

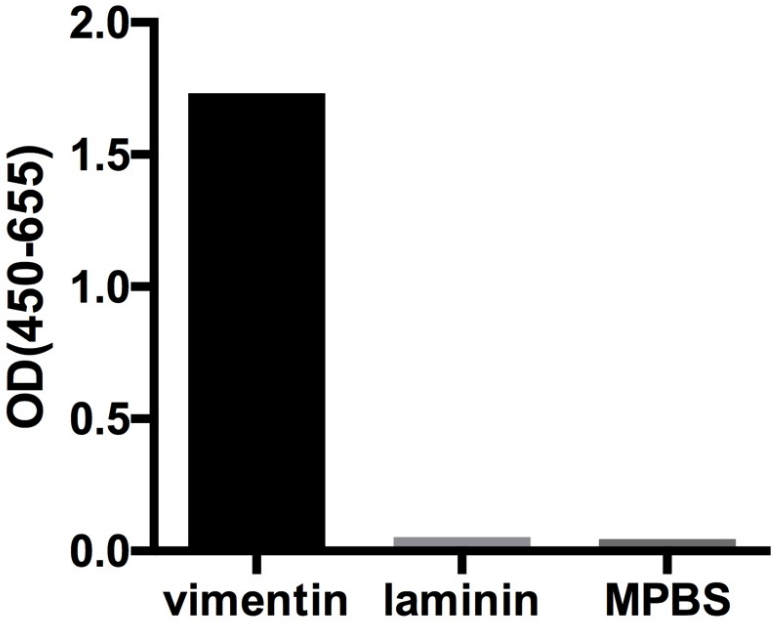

Supplementary Figure S3 The LOB7 phage antibody was tested against laminin. By phage antibody ELISA using LOB7 against vimentin and laminin in parallel, it was seen that LOB7 preferentially binds to vimentin

Supplementary Figure S4

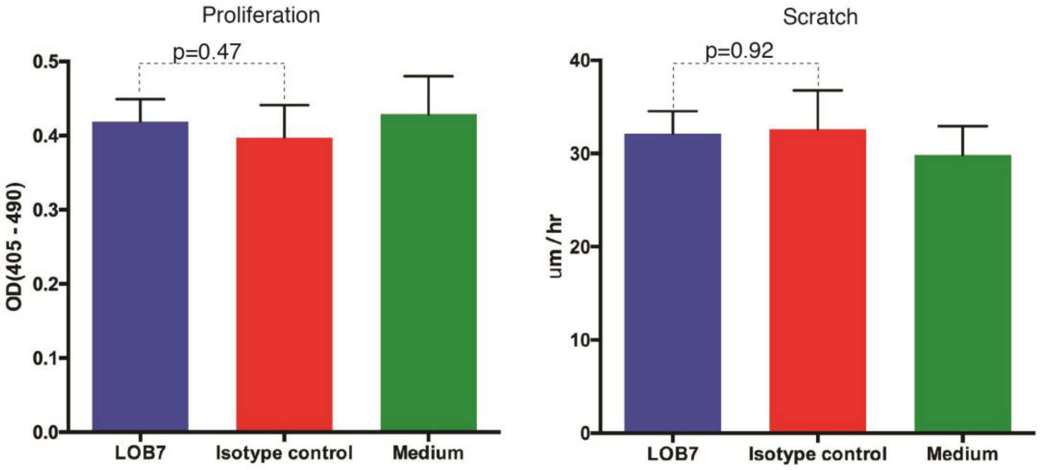

Supplementary Figure S4 LOB7 had no effect on the proliferation or the migration in the assays performed here. The error bars shown are the standard error of the mean of the experiments. The p values shown are calculated by a two tailed students t-test, assuming equal variance.

Supplementary figure S5

The microscopic images were automatically analysed using an in-house software package written in MathWorks Matlab (R2015a). To quantify the degree of tube formation (DoT) we used a standard morphological image processing method, known as skeletonisation. This method outputs a thinned version, i.e. a skeleton of the cell region surrounding the loops of cell free regions (figure s5). We subsequently used the total length of the branches spanning the skeleton as a measure of the DoT. As the number of loops grows, the skeleton develops more branches, which in turn increases the total length of the skeleton.

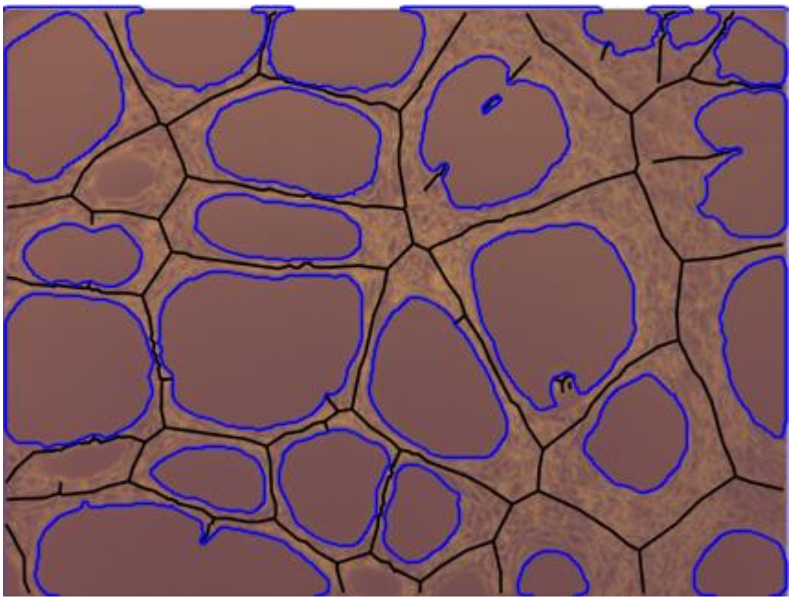

Supplementary Figure s5 Loops: Blue lines. Skeleton: Black lines

Skeletonisation requires a prior image segmentation step where the image is divided into regions corresponding to loops (blue contours in figure s5) and regions occupied by cells. For this purpose we used a standard segmentation technique based on Geodesic Active Contours.

Note that the skeletonisation process may produce many tiny branches if the boundaries between tissue and loops are not sufficiently smooth. These small branches represent a source of noise that is not related to the DoT. Consequently, to avoid overestimating the total skeleton length, all branches below a threshold length were discarded from the calculations. As a side effect, the skeleton may locally lose its connectivity but this is considered to have a negligible effect on the total skeleton length.
